# Agent-based modeling of urban exposome interventions: prospects, model architectures, and methodological challenges

**DOI:** 10.1093/exposome/osac009

**Published:** 2022-10-10

**Authors:** Tabea Sonnenschein, Simon Scheider, G. Ardine de Wit, Cathryn C. Tonne, Roel Vermeulen

**Affiliations:** 1 Human Geography and Spatial Planning, Faculty of Geosciences, Utrecht University, Utrecht, The Netherlands; 2 Julius Center for Health Sciences and Primary Care, University Medical Center Utrecht, Utrecht University, Utrecht, The Netherlands; 3 Institute of Risk Assessment Sciences, Utrecht University, Utrecht, The Netherlands; 4 Centre for Nutrition, Prevention and Healthcare, National Institute of Public Health and the Environment (RIVM), Bilthoven, The Netherlands; 5 Health Economics and Health Technology Assessment, Faculty of Science, Vrije Universiteit Amsterdam, Amsterdam, The Netherlands; 6 Barcelona Institute for Global Health, CIBER Epidemiologia y Salud Publica (CIBERESP), Universitat Pompeu Fabra (UPF), Barcelona, Spain

**Keywords:** urban exposome, agent-based modeling, social cost-benefit analysis, scenario modeling, urban health interventions, complex systems

## Abstract

With ever more people living in cities worldwide, it becomes increasingly important to understand and improve the impact of the urban habitat on livability, health behaviors, and health outcomes. However, implementing interventions that tackle the exposome in complex urban systems can be costly and have long-term, sometimes unforeseen, impacts. Hence, it is crucial to assess the health impact, cost-effectiveness, and social distributional impacts of possible urban exposome interventions (UEIs) before implementing them. Spatial agent-based modeling (ABM) can capture complex behavior-environment interactions, exposure dynamics, and social outcomes in a spatial context. This article discusses model architectures and methodological challenges for successfully modeling UEIs using spatial ABM. We review the potential and limitations of the method; model components required to capture active and passive exposure and intervention effects; human-environment interactions and their integration into the macro-level health impact assessment and social costs benefit analysis; and strategies for model calibration. Major challenges for a successful application of ABM to UEI assessment are (1) the design of realistic behavioral models that can capture different types of exposure and that respond to urban interventions, (2) the mismatch between the possible granularity of exposure estimates and the evidence for corresponding exposure-response functions, (3) the scalability issues that emerge when aiming to estimate long-term effects such as health and social impacts based on high-resolution models of human-environment interactions, (4) as well as the data- and computational complexity of calibrating the resulting agent-based model. Although challenges exist, strategies are proposed to improve the implementation of ABM in exposome research.

## Introduction

Stressing the fact that genetic models alone can only explain about 10% of human diseases and that environmental and behavioral factors are essential for their prevention, a new discipline called exposome science has emerged.1 The concept of the human exposome can be understood as “the totality of exposures we face throughout our lives and includes the food we ingest, the air we breathe, the objects we touch, the psychological stresses we face, and the activities in which we engage” that affect our health.2 On a global level, unhealthy food, followed by smoking, and air pollution are the risk factors with the largest attributable mortality.3 These environmental and behavioral factors belong to the most frequent causes of cardio-metabolic pulmonary diseases. The latter account for approximately one-third of all premature deaths globally.^4^

Acknowledging this impact of the built, physicochemical, and social environment on our health, either through exposure to environmental factors such as air pollution, noise, and heat or through impacting health-relevant behavior such as physical exercise, diet, or smoking, has considerable implications for spatial planning and architecture.^5,6^ In light of that, the growing trend of worldwide urbanization7 poses a threat but also an opportunity to design structural, risk-minimizing solutions to improve public health. We call urban interventions that target the human exposome to improve health “urban exposome interventions” (UEIs). Designing and implementing UEIs is challenging as they are often costly and will impact a city for many years and often in unforeseen ways. To avoid unintended social and health consequences and select the most cost-effective policy option, it is necessary to evaluate interventions ex ante, before implementing them. Understanding the social distributional impacts of interventions is moreover key for communicating who benefits and planning compensation for those who loose out. This can help build social acceptance of interventions.

While there are reviews of intervention effectiveness studies that are based on (quasi) experiments of implemented interventions,^8–13^ it is unknown whether in different cities, the same result would be achieved. The exact urban context and initial behavioral patterns influence how interventions are implemented and how they impact urban life. It can be expected that the effectiveness of interventions in the complex system of a city behaves in a non-linear fashion and that path dependency strongly impacts the outcomes. For example, constructing massive bike infrastructure in a very car-centered and polluted city might result in different changes of modal choice compared with constructing the same infrastructure in a city with already a majority of bikers. Apart from these real-world experiments, there are some health impact assessment (HIA) studies that evaluate hypothetical or real interventions,^14,15^ or the impact of a hypothetical change of behavior or an environmental stressor without intervention.^16–18^ The latter set of methods have limited capabilities to model behavioral and environmental feedback, adaptive processes, and non-linear effects. However, in many situations, such as in traffic, the behavior of one person depends on the behavior of others and may have externalities that affect others. In other words, these intervention assessment methods do not appropriately capture the complex interactions between the environment, human behavior, and health and social outcomes.^19^

In a nutshell, the complexity of UEI modeling has at least three dimensions: (1) The interaction between human behavior and the physical and social environment in cities^20^; (2) the health impact of the multiple, partially overlapping exposures resulting from an interaction of exposure and personal attributes^1,21^; and (3) the wider distributional social outcomes of interventions.^22^ Which method would be appropriate to estimate intervention impacts taking these interactions into account?

We and others have proposed using spatial agent-based modeling (ABM) to model the impacts of UEI scenarios. ABM is a simulation method in which autonomous agents (ie, representing citizens) interact with each other and the environment, changing the attributes of the agents and the environment. These microlevel interactions (transition rules) may ultimately impact macro-level outcomes, such as public health and social impacts. Spatial ABM means that the ABM model is embedded in geographic space represented by maps (eg, the urban environment and population distribution). ABM has been identified as one of few currently existing methods with an ability to capture complex processes, from emergence, feedback-loops to path dependency, non-linearity, and adaptation.^23,24^ We understand UEI-assessment ABMs as a class of models that aim to estimate changes in environmental stressors and individual behavior through UEIs, the resulting exposure interactions, their health, and social impacts using spatial ABM. There are a variety of scopes and contexts for which this class of model can be applied, which is why this article covers a range of different intervention types, exposure types, and potentially required sub-models.

While ABM has in recent years gained interest from the research community in a variety of disciplines also in public health,^25–31^ there is a lack of understanding of the model constraints and meaningful architecture for an UEI analysis. We aim to start filling that gap by reviewing the potential model specifications and methodological challenges when applying ABM to estimate impacts of UEIs and proposing strategies for overcoming them. Further, predictive ABMs require a sufficient understanding of the process underlying the model components. However, for some aspects of an UEI assessment ABMs, we are still lacking high-quality, standard process-based models, such as for human health behavior or granular exposure-dose-response functions as we will show in the following sections. This article contributes by identifying the necessary components and reviewing the state-of-the-art data and models for each of them, in order to direct future research. Further work is needed to analyze the level of detail required to capture impacts of UEIs and the modeling efficiencies that can be made without increasing uncertainty.

We discuss what ABMs have to offer in the context of UEI assessment and how this compares to quantitative HIA, and the model components required to capture the urban exposome and the effects of different types of UEIs. Subsequently, we elaborate on the concepts, spatial representation, sub-models, and transition functions that define the human-environment interactions in the model. Afterward, the integration of the various ABM modules—environmental stressors, behavior, acute health impacts, chronic health impacts, and the social cost-benefit analysis (SCBA)—and their temporal scales is discussed. Based on that, the emerging scalability issues and possible solutions are discussed. Next, the most efficient calibration strategies and necessary data sources are reviewed. The final section concludes with a discussion of the research agenda.

## The potential of spatial ABM for UEI assessment

### Prospects and constraints

There are multiple reasons why spatial ABM is a particularly suitable method for UEI modeling. (1) As environment-behavior interactions lie at the heart of ABMs, it is well-suited for modeling exposure influenced by specific behaviors, their timing, and location. An attractive feature is that it can be linked to geographic information systems (GIS) for representing spatial context.^30,32^ (2) Equally important is that any mathematical relationship—also non-linear, complex interactions—can be algorithmically modeled in an ABM (eg, the complex environment-behavior-health interactions discussed in the fourth paragraph of the “Introduction”). (3) ABMs can integrate many factors resulting in a holistic and context-dependent model.29 This also entails that it is possible to integrate a SCBA into the intervention assessment to analyze the health and economic consequences of UEIs for different social groups. (4) ABMs are fit for scenario modeling and forecasting, which allows estimating future effects contingent on interventions^32–35^ Since ABMs are based on process-oriented models of the components and relationships, a change in some of the input variables through an urban intervention (eg, an attribute of the infrastructure) will lead to different behavior of dependent components (eg, transport behavior). Sensitivity analysis, as well as quantitative uncertainty analysis, provides elegant ways of dealing with uncertainty. (5) Another important aspect of ABMs (and other simulation methods) is that it is an intuitive visualization and communication tool. ABMs can help explain the processes underlying intervention effects because they explicitly and thus transparently model relevant relations. ABM is thus different from a black-box approach to prediction. Moreover, by summarizing and displaying the effects of interventions, ABM has the potential to become a powerful tool to support decision-making (similar to other GIS-based planning support systems^36,37^) and participation processes.^38–40^ (6) Finally, once a suitable ABM framework has been built up in a generic, modular way, it can be (re-)used or extended for many purposes, such as new interventions, other cities, and improved calibration through newly available quality data, as has been done with the transport ABM MATSim.^41^

While these features are promising for UEI assessment, there are also limitations that need to be considered. The estimations of ABM will be only as good as the process-based models of which the ABM is composed. In other words, if the process underlying the model components and their interactions is not well understood or there is not enough data to appropriately calibrate its parameters, then the ABM might lead to a false sense of accuracy. Next, the number of variables and interactions can quickly lead to the curse of high-dimensionality,42 which can become very computationally and data-intensive to calibrate and validate.

### Benefits of ABM for HIA

Quantitative HIA is increasingly used for urban planning and has been recommended by the WHO.^43,44^ Urban Environmental HIA involves “modeling to determine likely exposures and epidemiological knowledge to translate these into estimates of potential health effects.”^45^ These estimations are usually based on deterministic or probabilistic models that integrate coefficients of former published studies.^14,17,18,46^ ABM can improve the modeling approaches on three dimensions: (1) calibration and use of data; (2) georeferencing; and (3) the ability to model complex interactions.

Regarding the calibration and use of data, deterministic and probabilistic HIAs model the changes in exposure caused by an intervention based on coefficients taken from generalized evidence (that combines multiple studies), if available.^14^ The problem is that there is no way to check if the coefficients are correct for the local context (eg, the influence of transport infrastructure on behavior). Generalized or average impacts might deviate substantially from the local impact because each neighborhood and location varies in relevant variables, such as socioeconomic context, demographics, urban built environment, climate, or culture. In an ABM, the causal relations or processes that give rise to a change in outcome are modeled. What is more, the transition functions that model the behavior of agents, the air pollution field, or any other element of the model can be calibrated using data from the place that is simulated. Various calibration strategies are discussed in Section 7. As the transition functions have been parameterized within the spatial context, the resulting predictions of behavior, environmental stressor, and exposure change are likely more realistic and robust.

The second dimension is the georeferencing of the model components. Deterministic and probabilistic HIAs usually use spatial data to determine the status quo environmental stressors and the difference caused by the intervention.^47,48^ Moreover, while most HIAs use a baseline health profile for the analyzed region as a whole, few use census or cohort data to identify the heterogeneous spatial distribution of risk groups. However, exposure is usually calculated by taking the residential address as a proxy for people’s 7*24 h location. A spatial ABM can model dynamic, interactive behavior in a concrete spatial environment, which might lead to more granular and accurate exposure and behavior estimations. Thereby, ABMs can differentiate between the spatial daily activity patterns of different social groups, for example, based on census and behavioral data. Finally, the exact way that an intervention, such as one in the transport system, is spatially implemented in a city has a difference in impact, as different people are affected by it, and different activities are offered in the place of intervention. An ABM can account for that contextdependency by integrating GIS layers of urban environmental features, a spatial layer of the intervention, and a heterogeneous population of residents with daily lifestyle patterns.

Concerning the third dimension, the ability to model complex interactions, probabilistic and deterministic models estimate the accumulated outcome of adaptive processes without modeling the underlying process. Particularly deterministic models do not model feedback and interaction, and variables are modeled in a static manner, except when explicitly modeling changes in the values. On the other hand, an ABM models causal relations, or heuristically approximated processes, that give rise to a change in value or behavior. Therewith, it predicts the non-linear or emergent outcome. The ability to explicitly model feedback and interaction is one of the core benefits that ABM can bring to HIA. This includes incorporating social interaction and adaptive behavior, such as the observation and imitation of others.

An ABM approach might benefit HIA particularly (1) when people’s behavior or spatial daily lifestyle patterns play an important role in the intervention outcome, (2) when incorporating behavioral feedback or complex environment-behavior interactions, or (3) when having to model interactions between dynamic spatial components. The following section dives more deeply into why human behavior is crucial for urban exposure.

## Identifying the required model components

### Urban exposure types

**Figure 1:**
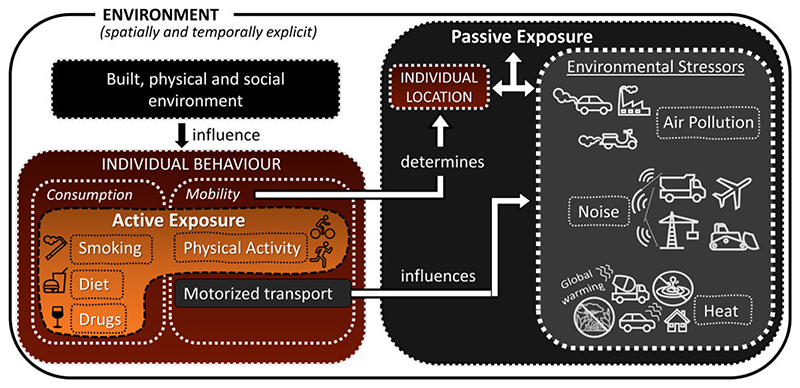
Urban exposure types and interactions.

To adequately model exposure in an ABM, we propose to distinguish between active or behavioral exposure, such as physical activity, smoking, and dietary habits, and passive or environmental exposure, such as all forms of air pollution and noise, as it has implications for modeling. We define active and passive exposures in terms of direct and indirect causal relations (see Figure 1). An active exposure is caused by individual behaviors, so that behavior is a mediator of the environment’s influence on active exposure (Environment) behavior) Active Exposure). (Arrows display causal relations, but they are not exhaustive and hence do not exclude the existence of other causal influences. For instance, there are more determinants of behavior than solely the environment.) Environment (eg, the quality of the cycling infrastructure and time of the day) plays only a role in influencing behavior (eg, an individual’s decision to cycle or not). We call this active, because the exposure is caused by a behavior. In contrast, passive exposure is directly caused by the environment (eg, air pollution concentration) at an individual’s location at a given time (Environment) Passive Exposure). Individual mobility behavior is only indirectly important insofar as it influences the location of the individual. This distinction between active and passive exposure is sometimes referred to as one between voluntary and involuntary exposures, one people have control over or not. However, next to this ethical significance, the distinction also has implications for the modeling requirements. While for passive exposure, tracking of people (mobility behavior) and environmental stressor concentrations need to be modeled in detail, for active exposure behavioral choices of humans need to be brought into focus. Figure 1 provides an overview of the types of exposures that are particularity salient in an urban environment and illustrates the role of people’s behavior for them. Since the mobility behavior of people influences also passive exposure, it can be argued that the ability of ABM to model complex environment-behavior interactions can improve the modeling of both active and passive exposure.

### UEIs and their modeling requirements

Cities (urban policy makers) have influence on a large set of factors that impact the Urban Exposome, such as the transport system, land use planning, and the design of public space, commercial regulation, and the housing market. An overview of major urban interventions assigned to these four domains, the exposures they target, and the model components that are central to their simulation can be found in Table 1. The interventions were collected from World Health Organization,^4^ Tonne et al.,^5^ Nieuwenhuijsen,6 Foster et al.,8 Freak-Poli et al.,^9^ Baker et al.,^10^ Burns et al.,11 Salam et al.,^12^ Freudenberg et al.,^49^ Münzel et al.,^50^ Donnelly et al.,51 Amorim et al.,^52^ Mueller et al.,^53^ and Peng et al.^54^ Note that most interventions target or at least affect multiple exposures. Different interventions require different behavioral and environmental model components (see last column of Table 1), but there are overlaps in the component requirements. To increase the impact and appreciate the full value of a developed model, one can model multiple interventions with similar modeling requirements (eg, multiple transport interventions) instead of only one. Mobility behavior is the most widely required type of behavior across the intervention types, because it is an indirect component of most passive exposures. Active exposure also requires modeling of other specific activity choices (eg, smoking, eating, and physical activity).

The decision for an intervention type to be evaluated needs to be taken early in the model building process. From a methodological perspective, modeling interventions requires that there is (1) data available for calibrating the required model components to ensure realistic scenarios as well as (2) the computational resources to model the required intervention components and interactions. After having decided on the intervention type (eg, see Table 1), an actual implementation of such an intervention has to be designed for a specific local context, potentially through a consultation of local planners or experts. Choices need to be made about the scale or magnitude (eg, spatial extent, number of features built, and regulation thresholds), the target group, and the period of implementation. There is a trade-off between the need for standardization and for realism of intervention modeling. Methodologically speaking, interventions need to be designed in a standardized way when wanting to compare cities and have a reproducible study. However, the more generic the intervention design is, the less realistic and hence the less useful its analysis for planning decisions within individual cities. Fortunately, one can test multiple intervention designs without having to remodel other ABM components. Multiple scenario modeling can moreover help understand which scope and form of an intervention has the best effects.

## Modeling human-environment interactions

To model the spatial context of interventions, model components representing the Urban Environment and human agents have to be georeferenced, resulting in multiple GIS layers interacting with each other. Decisions have to be taken concerning which components to model, how to conceptualize them in terms of spatial information (eg, as discrete object, continuous field, network, or event^55^), on what resolution levels to represent them and what operations to use for their interactions.^56^ While it is tempting to represent components and processes as granular and detailed as possible, the model has to remain verifiable. In other words, how detailed ABMs can become is limited by the resolution level of the available data and evidence.

### Spatiotemporal resolution and representation

Previous studies in environmental epidemiology have mostly used daily or annually average raster cell or statistical area data on environmental stressors and joined it with the residential location of study participants to identify passive exposure impacts.^50,57-59^ This method is based on the assumptions that people rarely leave their residential neighborhood and that the daily or annual average environmental stressor values of the raster cells or neighborhood units are a good proxy for the environment people are exposed to. However, the actual process of exposure is much more complex, dynamic, and granular as most individuals move between a wide range of indoor and outdoor environments on a daily basis. Studies show that ozone, temperature, and NOx concentrations strongly vary on an hourly basis and across space, and that when taking into account the movement trajectories, personal passive exposure estimates deviate substantially from the one estimated based on the residential address.^60,61^ Moreover, multiple studies have found that air pollution exposure calculated based on residential address causes an underestimation of the health effect compared with an exposure estimation taking time-activity patterns into account.^62–64^ One could argue that the epidemiology of exposure effects is not yet based on these granular estimations and that consequently it will be hard to base high-resolution exposure-response functions on evidence. However, it is possible to aggregate exposure estimations to a level where evidence for exposure-response functions can be found. Moreover, we increasingly have access to—or at least the ability to collect—hyperlocal data using, for example, wearable sensors to analyze spatiotemporal distributions or personalized exposure measurements of environmental factors, such as air pollution, noise, etc. with much greater resolution and precision.^65–67^ Data from information, communication, and Global Positioning System (GPS) technologies allow us to model or capture daily lifestyles and mobility patterns of a subpopulation in their specific environment, which much more closely approximates their actual exposure.^68^ There is a small but growing number of studies that have used wearable sensory devices, trackers, and time activity diaries to capture the specific personal exposure, which can be more accurate than the estimations based on aggregated data.^66,69-71^ While it is still difficult to collect data using sensors in large-scale health studies, it can be argued that it is only a matter of time until robust evidence on health effects of high-resolution exposure becomes more readily available.

A very good example of exposure estimations of high granularity in an ABM is the study of Chapizanis et al.30 The purpose of their ABM was to estimate personal exposure of the Thessaloniki (Greece) population, taking the spatial activity patterns and heterogeneous environmental stressor concentrations into account. They generated an agent population based on available spatial census data. To model the agents’ behavior, the authors used existing time activity data from the Harmonized European Time Use Survey (HETUS),72 which encodes the time use for activities in fixed 10-min time slots. Next, they used existing maps of hourly variations of PM2.5 and PM10 concentrations for each building block (30-40 m) that were pre-calculated using atmospheric dispersion models in the study of Sarigiannis et al.^73^ Indoor PM2.5 concentrations were estimated using the INTERA computational platform (INTERA, 2011). They further used GPS tracking data that were collected in the HEALS project and combined it with the air pollution maps to calibrate and validate the personalized exposure estimations of the model.

**Figure 2:**
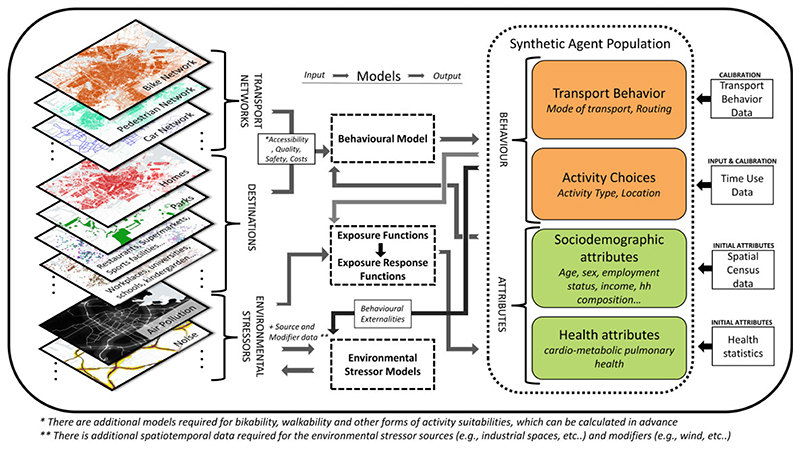
Human-environment interactions: components, models, and data.

The study of Chapizanis et al.^30^ is an ambitious and relevant example of how an Urban Exposure ABM can be embedded in spatial data to ensure realistic estimations. However, to be able to predict behavior and environmental change through urban interventions, we will have to go further (see Figure 2 for an overview of components required for human-environment interaction). If we want to capture feedback effects, we need to model the behavior dependent on the urban environment (eg, transport system, accessibility to, and quality of destinations) as well as individual and social factors. Next, the concentration of environmental stressors may need to be modeled dependent on the transport behavior of the agents and other source and modifier data.

One of the most crucial data inputs is the synthetic agent population. There is a variety of methods that can be used for generating a synthetic agent population and the choice depends on the available data.^74^ In general, combinatorial optimization methods^75^ can be used to duplicate real individual-level records, while synthetic reconstruction^76^ generates individual level agent attributes based on distributions of variables, such as widely available marginal and stratified distributions published by national censuses. Both groups of methods can be applied in such a way that multi-variable joint distributions are taken into account.

### Sub-models and transition functions

There are three groups of core sub-models characterizing human-environment interactions, whereby each contains a set of transition functions (see Figure 2): (1) the behavioral model; (2) the exposure and exposure-response functions, and (3) the environmental stressor models. The arrows in the figure display the input and output relations of the environmental and individuallevel attributes with the three models. A challenge that all models share is the minimization of uncertainty coming from the model specification. Specifically, the selection of variables and relationships to be represented has to be validated and the number of assumptions reduced. For that purpose, it makes sense to use existing evidence to justify the model structure, whereby the sources of evidence and the complexity of evidence extraction and synthesis vary between the different sub-models. Additionally, the parameters of the models have to be calibrated as we discuss in the last section. Finally, sensitivity analysis can be used to quantify the uncertainty coming from different model components. This section analyses the state-of-the-art models and sources for structural model validation for each of the submodels.

The core requirement of the behavioral model is that it should capture realistic health-relevant behavior (transport and activity choices) dependent on the urban environment and individual attributes, so that it can predict behavior change through an urban intervention. While plenty of different generalized cognitive architectures have been developed in the field of artificial intelligence (eg, ACT/R,77 SOAR,^78^ CLARION,^79^ or for a comprehensive review^80^), they are not designed to simulate a whole city of agents and either have redundant cognitive functions (like speech, motor skills) or lack important representations, such as social norms. There has also been a plethora of different ABM applications with unique, model-specific behavioral frameworks that do not meet our model requirements.^30,33,35,81-83^ The most useful behavioral frameworks that could be specified and amended for our application purpose are behavioral architectures developed in the field of multi-agent systems, such as the beliefs-desires- intentions architecture and its derivatives^84,85^ or the normative-agents framework.^86^ However, these frameworks are rather abstract in the sense that they do not inform the selection of variables and data needed to model a specific type of behavior. Hence, the exact specification and application to UEI assessment remains a non-trivial task. As a possible starting point, we suggest utilizing published empirical evidence on significant behavior determinants and interactions in order to inform the variable selection and structure of behavioral models. Considering the vastness and complexity of the behavior science literature, perhaps natural language processing and machine learning can be used to automatize part of the knowledge extraction process.

Some of the transition functions that will govern the influence of the environment on behavior can already be identified. The environment influences decision-making via perception. Perceivable features of the environment that may be part of behavioral transition functions are the (a) accessibility of destinations, such as healthy food stores, sports facilities, a subway station, etc., the (b) suitability of available destinations or routes for certain activities, for example, the walkability, bikeability, affordability, or safety, or the (c) visibility of other people engaging in a specific activity. Studies on how environmental factors influence health relevant behavior should function as a basis for modeling these relations.^87–90^

The second group of models are the exposure and exposure-response functions. To estimate the dose of a passive exposure, a simple spatial join of the agent’s location or route at the time-step of the environmental stressor map is sufficient. Additionally, other individual-level variables can be part of the individual exposure dose calculation depending on the exposure pathway: inhalation, ingestion, mechanosensation, or thermoception. For example, inhalation rates (dependent on age, body weight, and activities) and tidal volume could be relevant for air pollution,^91^ while hearing sensitivity and noise annoyance could be included in the noise dose, etc. However, actual inclusion of such factors will depend on the availability of health studies which cover these individual differences. For active exposure, the duration and intensity of physical activity or the quantity of health-relevant substances consumed (food, alcohol, etc.) should be captured.

The exposure and dose estimated by the exposure functions will be the input for the exposure-response functions that alter the human health levels either temporarily or permanently. Hereby, the exposure levels have to be aggregated to a temporal period and metric for which robust exposure-response functions are available. This would be hours to weeks for acute health impacts and one to several years for chronic health impacts and could be concentration or dose based. In the future, evidence of Omics studies might inform heterogeneous exposure-response functions and measures of susceptibility.

The final set of models are the environmental stressor models. The last decades have seen a growth and improvement of process-oriented models and even tools for estimating environmental stressors, which can be either directly integrated or translated into an ABM. Since these models are based on deterministic physical knowledge, their structure will not have to be validated again. To model different types of air pollution, a set of atmospheric dispersion models are available.^73,92-94^ To model urban noise, multi-source noise diffusion models and mapping tools have been developed.^95–99^ Finally, for dynamically estimating temperature, heat transfer models are increasingly used to assess the urban heat island effect.^100,101^ For certain environmental stressors (eg, noise and air pollution), the behavior of an agent (eg, driving with fuel-based transport) can have externalities that affect the environmental stressor field.102 In that case, the agent’s behavior will become a source within the environmental stressor model.

## Modules integration, temporal scales, and outcome variables

The ABM model consists of different modules that are to a large extent self-contained but interact with each other: The environmental stressors, the urban perceivable environment (eg, transport networks and destinations), agents and their behavior, the acute and chronic health impacts, and the social costs and benefits. The simulations within and between these modules have to be aggregated to arrive at estimations of the effects of the UEI. A challenge particular to evaluating long-term outcomes (such as social costs and benefits and chronic health impacts) with a high-resolution ABM is the integration of various temporal resolution levels and extent requirements.

The most granular modules are the environmental stressors, the perceivable environment, and behavior as discussed in the last section. The smallest time-steps in which variables belonging to these modules should be able to change are 10 min-1 h, since behavioral activities are likely to change in the course of an hour and that is the resolution for which time-use data are available, for example, from HETUS. Given that environmental stressors depend on agent behavior, their temporal resolution should be harmonized. The perceived environment is mostly static, but the few elements that are dynamic and relevant for behavior (eg, weather and seasonal changes of greenery) should also be able to change with the same temporal resolution as the agent’s behavior. The interaction of environmental stressors and the momentary location of agents (passive exposure) and the health relevant behavior (active exposure) are used to compute the exposures that are the input for the exposure-response functions. The acute health impacts should be calculated in time-steps of either a couple of hours or weeks, while the chronic health impacts should be calculated every one to several years. Chronic impacts accumulate over a lifetime, which is also the temporal extent for which they need to be assessed.

Finally, the module with the lowest resolution is the SCBA, which incorporates the health impact variables generated by the exposure-response functions with costs and benefits of UEIs, preferably for different social groups. The SCBA compares the health impacts and total societal costs of different intervention scenarios with a baseline scenario, usually one in which no interventions are implemented and the status quo does not change. The calculation includes the costs of the intervention itself and the returns of the investment (eg, the number of prevented diseases and healthcare savings). Some of the most important returns of the investments will be changes in mortality, quality-adjusted life years (QALYs), wellbeing-adjusted life years (WALYs), and disability-adjusted life years (DALYs). In case an UEI does not cause much of a change in these outcomes due to scale of the intervention area or observational period, it is also possible to evaluate its impact on active and passive exposure (health behaviors and environmental stressor exposure). The social groups for the analysis of distributional effects can be defined based on any of the individual agent variables, such as ages, socio-economic status, or initial health conditions. Moreover, different spatial groups, such as neighborhoods can be compared. SCBA involves a lot of global variables which will not be explicitly modeled in form of process-based models.

### Scalability challenges and possible solutions

Mathematically speaking, it is possible to integrate input variables of any of the higher resolution modules into lower resolution modules (like the SCBA) through aggregation, averaging, boolean conditioning, or any other arithmetic operation. However, there is a tension between the goal of modeling a high-resolution human-environment simulation and the requirement of a large temporal extent over which this simulation needs to run: The combination of these two goals leads to a computational complexity problem. With 10 min time-steps, one would require 3.942.000 time-steps to run through a lifetime of 75 years. Moreover, ABMs incorporate certain degrees of randomness or uncertainty, necessitating an average over multiple runs (the Monte Carlo method) to achieve a more robust result. This needs to be done for a reference or baseline scenario and an intervention scenario, to be able to identify the difference. The model outcome variability across runs is indicative of the sensitivity of the model to the sampled and random variables and a means to quantify the uncertainty coming from sampling variability. Additionally, bootstrapping^103,104^ can be used to get a more detailed understanding of the sampling variability without computationally expensive additional runs. To obtain a scalable computational model, we need to avoid simulation of redundant parts and unnecessary detail.

There are several strategies that help reducing the computational load apart from decreasing the granularity of the highresolution modules.

- Extrapolation: When intervention impacts on behavior and environmental stressors and exposure begin to stagnate, it is not necessary to model a whole lifetime with a high-resolution ABM. In this case, one can simulate the granular daily behavior and environmental interactions until no significant change apart from normal fluctuations can be found anymore. From then on the future exposure, health impacts and social costs and benefits can be extrapolated for the rest of a lifetime. The reliability of such extrapolation strategies can be tested by comparing results to the ones of full simulations over a lifetime.
- Aggregation: Even though behavioral activities (trips, certain activities) may change with a rate of 10-20 min, it may not be necessary to model behavioral decisions in such time steps. Instead, one can have agents plan a sequence of activities over a period given by a coarser time step (eg, 1 h). In this way, one can aggregate exposures resulting from the spatial interaction of the agents and the environment over 1 h without loosing high-resolution spatiotemporal variation. This allows to take much larger time-steps in the ABM simulation, making it more scalable.

Finally, one can reduce the general computational load by random sub-sampling of the agent population to estimate intervention effects, and by parallelizing computation across central processing units or graphics processing units.

## State-of-the-art calibration strategies and data

A core challenge of ABM is to find the parameters for the behavioral and interaction rules of the agents in the model so that it best reflects real-world observations.^105^ A simulation model is most useful when it is realistic, because only then it can generate reliable estimations as a basis for policy decisions.^24^ Calibrating the model takes four steps: (1) the selection of the parameters to be calibrated, (2) the identification and preparation of required data, (3) the definition of an objective function that reflects the level of agreement between the modeled output and the observed data, and (4) a method for optimizing the search for the best parameter values.

The core challenge in the first step is the fact that ABMs usually contain many model components and interactions and it is nearly impossible to parameterize all model components due to lack of data, time, and computational resources. We therefore need to prioritize the model components and corresponding parameters based on their relevance for the goal of the simula-tion.^42^ Parameters regarding environmental stressors and agent behavior are most important for exposure estimations and should therefore be prioritized in the calibration process. In order to avoid overestimation of parameter importance, one should calibrate correlated variables that influence the same outcome at the same time. In case of a strong correlation without additional functionality one should exclude redundant parameters and corresponding variables. The last column in Table 1, which lists the core components, can serve as a reference for finding the most important parameters for calibration depending on the intervention. Moreover, sensitivity analysis, a statistical method that quantifies changes in output through variations in input, can help to rank the importance of parameters for calibration.^105^

Having identified the parameters, one needs to find or collect the data needed for calibration. To calibrate environmental stressor models, one needs measurement data of the environmental stressors with the same or higher spatiotemporal resolution as the ABM from the same city. Both measurement points or aggregated raster data work, but the more complete the coverage of the ABMs spatial extent and the more temporal coverage of seasons, the more representative the distribution for calibrating parameter settings. For the calibration of the behavioral model, transport behavior data (modal choice, routes, trip length, etc.), and time-use surveys that are spatially explicit and capture socio-demographic attributes of individuals are required. Once georeferenced, the datasets can be spatially joined to data about the built-environment. The socio-demographic attributes will be needed for matching ABM agents to similar individuals of the observed datasets. While both the environmental stressor data and the behavioral data are not ubiquitous, it can be found for most cities within the EU. While EU environmental and health regulation forces cities and nation states to monitor different types of environmental stressors (even though not all), HETUS provides access to national time-use surveys. Many countries moreover conduct their own transport surveys.

The third step requires selecting a measure of model fitness in consideration of the model goal. This is crucial as the quality of the calibration is dependent on how useful and specific the fitness measure is.^23^ A core challenge in that step comes from the fact that ABMs often operate on multiple hierarchical levels, which makes it particularly difficult to compare outputs to observations of the underlying system.^24^ For example, one could compare the output of a parameterized transport behavior model to the behavioral patterns of individuals but also to the traffic volume in each raster cell or even the whole city, which might lead to discrepant optimal parameter settings. Some proposals to overcome this problem have been: (1) combining multiple objective functions^42^ and (2) pattern-oriented modeling (for further information, see Grimm et al.^106^).

In the fourth step, the search for the parameters that lead to the closest fit between the model outcomes and the observations needs to be optimized. Certain methods allow to avoid a computationally intensive brute force run through all possible parameter combinations to evaluate their performance. One of the most efficient optimization methods is the hill-climbing technique, which builds on the principle of incremental change toward the optimum. Its most efficient version is based on direct search algorithms, which aim to find improved objective function values by searching along trial directions from the current point.^42^ Finding only a local minimum can be avoided by starting the algorithm from varied points in the parameter space and checking the consistency of the final sets of parameter values.^42^ Some more sophisticated parameter search methods are based on genetic algorithms, such as particle swarm optimization,^107^ in which sets of behavior defining rules evolve to increase their model fitness until a global optimum fitness is reached.^23,42^ Evolutionary Monte Carlo goes even further by incorporating attractive features of simulated annealing and genetic algorithms into a framework of Markov chain Monte Carlo.^108–110^ Some more recent calibration optimization strategies are based on surrogatemodeling or meta-modeling to shortcut the ABM simulation process. Hereby, a computationally cheap approximation of the real model is generated based on a representative sample of parameter space values, using, for example, Kriging111 or supervised machine learning.^112^

## Discussion and conclusion

In this article, we have discussed the prospects, value, concepts, and methods for implementation and associated challenges of simulating UEIs using ABM integrated with a SCBA. To sum up, ABM has the potential to become a powerful tool for robust estimations of health impacts and social costs of UEIs. It is a bottom-up modeling method that fully exploits state-of-the-art process-based models for environmental stressors (eg, air pollution and noise), spatiotemporal data of our environment and lifestyle patterns, and the knowledge on behavioral determinants. By integrating causal relationships on several environmental and behavioral levels and by calibrating parameters in an urban context, estimations of intervention effects are likely to be of higher quality than in previously used approaches. ABM allows modeling the complex environment-behavior interactions, health impacts, and social distributional impacts in an integrated manner.

However, there are still some challenges on our way toward successfully exploiting ABM for UEI assessment (see box containing research agenda below). Firstly, it remains a non-trivial task to design a realistic, parameterizable behavioral model that is able to capture different types of exposures and responds to urban interventions. Secondly, there is a mismatch between the possible granularity of exposure estimations and the available epidemiological evidence needed for corresponding exposure-response functions, which limits the spatiotemporal resolution that an ABM can meaningfully explore. The third challenge relates to the scalability problem and computational complexity that emerge when aiming to estimate long-term effects such as health and social impacts based on high-resolution models of human-environment interactions. Finally, more work is needed to understand how to calibrate the parameters of a highdimensional UEI ABM most efficiently. In this article, we have discussed basic strategies and design options for tackling these challenges. However, it remains for future research to specify, implement, and test them. Further, future work should analyze the level of detail that is needed to capture impacts of UEIs and the short-cuts that can be made without increasing uncertainty.

Even if some of these challenges remain unsolved, the ABM forces us to investigate the processes and changes induced by an UEI. If successful, it gives us good approximations of intervention impacts. The latter can be tested by comparing estimations (eg, behavior and exposure change) of an UEI ABM model that was calibrated with data from before a real-world intervention to the actual impact of the intervention once implemented.

## Figures and Tables

**Table 1:** UEIs and model requirements

Domain	Intervention	Target exposures	Core model components
Transport	Car-free zones Bike infrastructure/sharing Pedestrian infrastructure Green walking and biking routes, separate from sources of pollution Removing car infrastructure/increase parking prices Subway/tram expansion	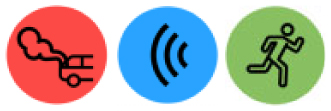	Modal choice; destination choice; car emissions; traffic noise; routing
	Speed limits Greening public transport	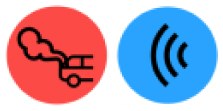	Modal choice; destination choice; car emissions; traffic noise; routing
	Low-emission zones	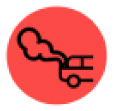	Modal choice; destination choice; car emissions; routing
	Subsidizing/expanding e-mobility and infrastructure	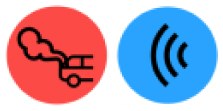	Vehicle buying choice; traffic noise; car emissions; modal choice; destination choice; routing
Land Use and Public Space	Green spaces	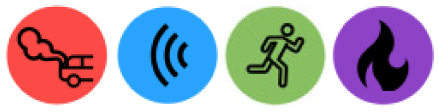	Noise and air pollution filtering of vegetation; temperature; use of green spaces; mobility
	Mixed land-use/accessibility to work and amenities	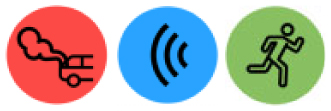	Destination choice; routing; modal choice; car emissions; traffic noise
	Urban gardening	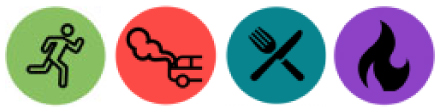	Gardening activity; eating; air pollution; temperature; mobility
	Public sports facilities	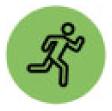	Sports behavior
	Blue spaces	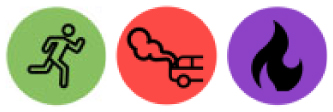	Use of blue spaces; air pollution; temperature; mobility
	Relocating industry	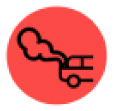	Industry emissions; mobility
	More trees and vegetation	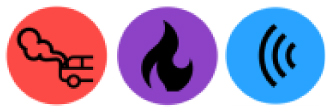	Noise and air pollution filtering of vegetation; temperature; mobility
	Designated smoking spaces	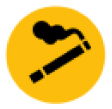	Smoking behavior; mobility
	Changing building and road materials	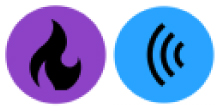	Mobility; heat and noise absorption of built environment
Commercial Regulation	Limiting fast food vending points Farmers and fresh food markets	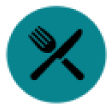	Restaurant/market patronage; eating behavior
	Limiting vending points for alcohol	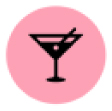	Alcohol store patronage; drinking
	Prohibiting smoking in bars and inside public spaces	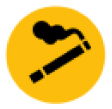	Inside public space and bar patronage; smoking behavior
Housing	Building health and safety standards	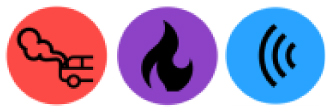	Residential pollution; residential noise; residential heat; mobility
	Ban on residential coal/wood burning Subsidizing stove exchange	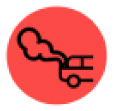	Residential pollution; mobility
	Subsidizing green roofs	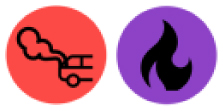	Temperature, air pollution filtering of vegetation; mobility

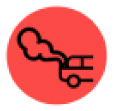
 air pollution, 
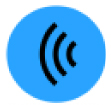
 noise, 
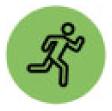
 physical activity, 
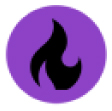
 heat,
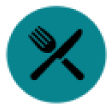
 diet, 
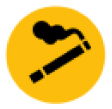
 smoking (passive and active), and 
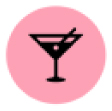
 alcohol.

## Data Availability

No new data were generated or analyzed in support of this research.

## Research Agenda for implementing UEI ABMs

**Environment, behavior, and their interaction**

- How to modularize and integrate existing models of environmental stressors within an ABM?
- How to model behavior of agents adequately to capture active and passive exposure and predict behavior change through urban interventions?
- What spatial components do we need to incorporate in the model and what data and concepts are required to capture and represent them?
- What spatial operations do we use for modeling spatial interactions?
- What temporal and spatial resolution levels are most adequate for capturing the variables?

**Exposure-response functions**

- How can granular exposure estimates resulting from an UEI ABM be matched with epidemiological evidence for risk functions?
- How can we reliably model heterogeneous risk functions across the population?

**Interventions**

- How to model realistic interventions?
- How to formalize the intervention designs to allow reproduction, cross-comparison with another city?
- How can the ABM be built so that multiple interventions can be tested with it?

**Scaling and calibration**

- How can the macro-level SCBA and health impact results be most efficiently derived from the micro-level UEI ABM?
- What level of detail is needed to accurately estimate exposures? Where can short-cuts be made without increasing uncertainties in the outputs?
- What are adequate strategies for scalability?
- How can we test and validate transportability of the model to other cities?
- What are meaningful measures of model fitness?
- What are the best methods for calibrating an UEI ABM?

## References

[1] Wild CP (2005). Complementing the genome with an “exposome”: the outstanding challenge of environmental exposure measurement in molecular epidemiology. Cancer Epidemiol Biomarkers Prev.

[2] Miller GW, Miller GW (2014). The Exposome: A Primer.

[3] Stanaway JD, Afshin A, Gakidou E (2018). Global, regional, and national comparative risk assessment of 84 behavioural, environmental and occupational, and metabolic risks or clusters of risks for 195 countries and territories, 1990-2017: a systematic analysis for the Global Burden of Disease Study 2017. Lancet.

[4] World Health Organization (2014). Global Status Report on Noncommunicable Diseases 2014.

[5] Tonne C, Adair L, Adlakha D (2021). Defining pathways to healthy sustainable urban development. Environ Int.

[6] Nieuwenhuijsen MJ (2020). Urban and transport planning pathways to carbon neutral, liveable and healthy cities; a review of the current evidence. Environ Int.

[7] UN Department of Economic and Social Affairs (2018). World Urbanization Prospects.

[8] Foster C, Hillsdon M, Thorogood M, Kaur A, Wedatilake T, Cochrane Heart Group (2005). Interventions for promoting physical activity. Cochrane Database Syst Rev.

[9] Freak-Poli RL, Cumpston M, Albarqouni L, Clemes SA, Peeters A (2020). Workplace pedometer interventions for increasing physical activity. Cochrane Database Syst Rev.

[10] Baker PR, Francis DP, Soares J, Weightman AL, Foster C (2015). Cochrane Public Health Group. Community wide interventions for increasing physical activity. Cochrane Database Syst Rev.

[11] Burns J, Boogaard H, Polus S (2019). Interventions to reduce ambient particulate matter air pollution and their effect on health (Review). Cochrane Database Syst Rev.

[12] Salam RA, Das JK, Irfan O, Ahmed W, Sheikh SS, Bhutta ZA (2020). Effects of preventive nutrition interventions among adolescents on health and nutritional status in low- and middle-income countries: A systematic review. Campbell Syst Rev.

[13] Nieuwenhuijsen MJ (2018). Influence of urban and transport planning and the city environment on cardiovascular disease /692/4019 /692/499 review-article. Nat Rev Cardiol.

[14] Waheed F, Ferguson GM, Ollson CA, MacLellan JI, McCallum LC, Cole DC (2018). Health impact assessment of transportation projects, plans and policies: a scoping review. Environ Impact Assess Rev.

[15] Fischer TB, Fawcett P, Nowacki J, Clement S, Hayes S, Jha-Thakur U (2018). Consideration of urban green space in impact assessments for health. Impact Assess Proj Apprais.

[16] Khomenko S, Cirach M, Pereira-Barboza E (2021). Premature mortality due to air pollution in European cities: a health impact assessment. Lancet Planet Health.

[17] Woodcock J, Edwards P, Tonne C (2009). Public health benefits of strategies to reduce greenhouse-gas emissions: urban land transport. Lancet.

[18] Hartog JJ, Boogaard H, Nijland H, Hoek G (2010). Do the health benefits of cycling outweigh the risks?. Environ Health Perspect.

[19] Rydin Y, Bleahu A, Davies M (2012). Shaping cities for health: complexity and the planning of urban environments in the 21st century. Lancet.

[20] Batty M (2013). The New Science of Cities.

[21] Tonne C, Basagana X, Chaix B (2017). New frontiers for environmental epidemiology in a changing world. Environ Int.

[22] Ham M, Manley D, Bailey N, Simpson L, Maclennan D (2012). Neighbourhood Effects Research: New Perspectives.

[23] Railsback SF (2001). Concepts from complex adaptive systems as a framework for individual-based modelling. Ecol Model.

[24] Crooks A, Heppenstall A, Malleson N (2017). Agent-Based Modeling.

[25] Marshall BD, Galea S (2015). Formalizing the role of agent-based modeling in causal inference and epidemiology. Am J Epidemiol.

[26] Tracy M, Cerda M, Keyes KM (2018). Agent-based modeling in public health: current applications and future directions. Annu Rev Public Health.

[27] Li Y, Lawley MA, Siscovick DS, Zhang D, Pagan JA (2016). Agent-based modeling of chronic diseases: a narrative review and future research directions. Prev Chronic Dis.

[28] Auchincloss AH, Diez Roux AV (2008). A new tool for epidemiology: the usefulness of dynamic-agent models in understanding place effects on health; Am. J Epidemiol.

[29] Badham J, Chattoe-Brown E, Gilbert N, Chalabi Z, Kee F, Hunter RF (2018). Developing agent-based models of complex health behaviour. Health Place.

[30] Chapizanis D, Karakitsios S, Gotti A, Sarigiannis DA (2021). Assessing personal exposure using agent based modelling informed by sensors technology. Environ Res.

[31] Maglio PP, Mabry PL (2011). Agent-based models and systems science approaches to public health. Am J Prev Med.

[32] Almagor J, Martin A, McCrorie P, Mitchell R (2021). How can an agentbased model explore the impact of interventions on children’s physical activity in an urban environment?. Health Place.

[33] Hennessy E, Ornstein JT, Economos CD (2016). Designing an agent-based model for childhood obesity interventions: a case study of childobesity180. Prev Chronic Dis.

[34] Oh S, Seshadri R, Azevedo CL, Kumar N, Basak K, Ben-Akiva M (2020). Assessing the impacts of automated mobility-on-demand through agent-based simulation: a study of Singapore. Transport Res A Policy Pract.

[35] Lu M, Hsu SC, Chen PC, Lee WY (2018). Improving the sustainability of integrated transportation system with bike-sharing: a spatial agent-based approach. Sustain Cities Soc.

[36] Pelzer P, Geertman S, Heijden R, Rouwette E (2014). The added value of planning support systems: a practitioner’s perspective. Comput Environ Urban Syst.

[37] Maarseveen M, Martinez J, Flacke J (2018). GIS in Sustainable Urban Planning and Management: A Global Perspective.

[38] Zellner ML, Lyons LB, Hoch CJ, Weizeorick J, Kunda C, Milz DC (2012). Modeling, learning, and planning together: an application of participatory agent-based modeling to environmental planning. URISAJ.

[39] Seidl R (2015). A functional-dynamic reflection on participatory processes in modelingprojects. Ambio.

[40] Mehryar S, Schwarz N, Sliuzas R, Maarseveen M, Verhagen H, Borit M, Bravo B, Wijermans N (2020). Advances in Social Simulation: Looking in the Mirror Springer Proceedings in Complexity.

[41] Horni A, Nagel K, Axhausen KW (2016). The Multi-Agent Transport Simulation MATSim.

[42] Beven KJ (2002). Rainfall-Runoff Modelling: The Primer.

[43] WHO European Centre for Health Policy (1999). Health Impact Assessment—Main Concepts and Suggested Approach. Gothenburg Consensus Paper.

[44] WHO (2022). Health Impact Assessment.

[45] Briggs DJ (2008). A framework for integrated environmental health impact assessment of systemic risks. Environ Health.

[46] De Nazelle A, Nieuwenhuijsen MJ, Anto JM (2011). Improving health through policies that promote active travel: a review of evidence to support integrated health impact assessment. Environ Int.

[47] Mueller N, Rojas-Rueda D, Khreis H (2020). Changing the urban design of cities for health: the superblock model. Environ Int.

[48] Iungman T, Khomenko S, Nieuwenhuijsen M (2021). The impact of urban and transport planning on health: assessment of the attributable mortality burden in Madrid and Barcelona and its distribution by socioeconomic status. Environ Res.

[49] Freudenberg N, Galea S, Vlahov D (2005). Beyond urban penalty and urban sprawl: back to living conditions as the focus of urban health. J Community Health.

[50] Munzel T, Sprensen M, Gori T (2017). Environmental stressors and cardio-metabolic disease: part I—epidemiologic evidence supporting a role for noise and air pollution and effects of mitigation strategies. Eur Heart J.

[51] Donnelly D, Everard M, Chang AB (2006). Cochrane Airways Group. Indoor air modification interventions for prolonged nonspecific cough in children. Cochrane Database Syst Rev.

[52] Amorim JH, Valente J, Cascao P (2013). Pedestrian exposure to air pollution in cities: modeling the effect of roadside trees. Adv Meteorol.

[53] Mueller W, Steinle S, Parkka J (2020). Urban greenspace and the indoor environment: pathways to health via indoor particulate matter, noise, and road noise annoyance. Environ Res.

[54] Peng J, Liu Q, Xu Z (2020). How to effectively mitigate urban heat island effect? A perspective of waterbody patch size threshold. Landsc Urban Plan.

[55] Allen C, Hervey T, Lafia S, Phillips DW, Vahedi B, Kuhn W, Miller J, O’Sullivan D, Wiegand N (2016). Lecture Notes in Computer Science (Including Subseries Lecture Notes in Artificial Intelligence and Lecture Notes in Bioinformatics);9927 LNCS of Lecture Notes in Computer Science.

[56] Burrough PA, McDonnell RA, Lloyd CD (2015). Principles of Geographical Information Systems.

[57] Lu F, Xu D, Cheng Y (2015). Systematic review and meta-analysis of the adverse health effects of ambient PM2.5 and PM10 pollution in the Chinese population. Environ Res.

[58] Anderson H, Atkinson R, Peacock J, Marston L, Konstantinou K (2004). Meta-analysis of time-series studies and panel studies of particulate matter (PM) and ozone (O3).

[59] Chen H, Burnett RT, Kwong JC (2014). Spatial association between ambient fine particulate matter and incident hypertension. Circulation.

[60] Park YM, Kwan MP (2017). Individual exposure estimates may be erroneous when spatiotemporal variability of air pollution and human mobility are ignored. Health Place.

[61] Blanchard O, Deguen S, Kihal-Talantikite W, Francois R, Zmirou-Navier D (2018). Does residential mobility during pregnancy induce exposure misclassification for air pollution?. Environ Health.

[62] Dhondt S, Beckx C, Degraeuwe B (2012). Health impact assessment of air pollution using a dynamic exposure profile: implications for exposure and health impact estimates. Environ Impact Assess Rev.

[63] Setton E, Marshall JD, Brauer M (2011). The impact of daily mobility on exposure to traffic-related air pollution and health effect estimates. J Expo Sci Environ Epidemiol.

[64] Ragettli MS, Phuleria HC, Tsai MY (2015). The relevance of commuter and work/school exposure in an epidemiological study on traffic-related air pollution. J Expo Sci Environ Epidemiol.

[65] Jovasevic-Stojanovic M, Bartonova A, Topalovic D, Lazovic I, Pokric B, Ristovski Z (2015). On the use of small and cheaper sensors and devices for indicative citizen-based monitoring of respirable particulate matter. Environ Pollut.

[66] Ueberham M, Schlink U (2018). Wearable sensors for multifactorial personal exposure measurements—a ranking study. Environ Int.

[67] Nieuwenhuijsen MJ, Donaire-Gonzalez D, Foraster M, Martinez D, Cisneros A (2014). Using personal sensors to assess the exposome and acute health effects. Int J Environ Res Public Health.

[68] Korpilo S, Virtanen T, Lehvavirta S (2017). Smartphone GPS tracking—inexpensive and efficient data collection on recreational movement. Landsc Urban Plan.

[69] Dons E, Rojas-Rueda D, Anaya-Boig E (2018). Transport mode choice and body mass index: cross-sectional and longitudinal evidence from a European-wide study. Environ Int.

[70] Ekblom-Bak E, Hellenius ML, Ekblom O, Engstrom LM, Ekblom B (2010). Independent associations of physical activity and cardiovascular fitness with cardiovascular risk in adults. Eur J Cardiovasc Prev Rehabil.

[71] Mila C, Salmon M, Sanchez M (2018). When, where, and what? Characterizing personal PM2.5 exposure in periurban India by integrating GPS, wearable camera, and ambient and personal monitoring data. Environ Sci Technol.

[72] Eurostat (2021). Harmonised European Time Use Survey (HETUS).

[73] Sarigiannis DA, Kontoroupis P, Nikolaki S, Gotti A, Chapizanis D, Karakitsios S (2017). Science of the total environment benefits on public health from transport-related greenhouse gas mitigation policies in Southeastern European cities. Sci Total Environ.

[74] Chapuis K, Taillandier P, Drogoul A (2022). Generation of Synthetic Populations in Social Simulations: A Review of Methods and Practices. JASSS.

[75] Williamson P, Birkin M, Rees PH (1998). The estimation of population microdata by using data from small area statistics and samples of anonymised records. Environment and Planning A: Economy and Space.

[76] Wilson AG, Pownall CE (1976). A new representation of the urban system for modelling and for the study of micro-level interdependence. Area.

[77] Byrne D (2000). The ACT-R/PM Project. AAAI Technical Report FS-00-03.

[78] Laird JE, Newell A, Rosenbloom PS (1987). SOAR: an architecture for general intelligence. Artif Intell.

[79] Sun R, Chipman SEF (2017). The Oxford Handbook of Cognitive Science.

[80] Balke T, Gilbert N (2014). Do agents make decisions?. JASSS.

[81] Brandon N, Dionisio KL, Isaacs K (2020). Simulating exposure-related behaviors using agent-based models embedded with needs-based artificial intelligence. J Expo Sci Environ Epidemiol.

[82] Plakolb S, Jager G, Hofer C, Fullsack M (2019). Mesoscopic urbantraffic simulation based on mobility behavior to calculate NOx emissions caused by private motorized transport. Atmosphere.

[83] Orr MG, Kaplan GA, Galea S (2016). Neighbourhood food, physical activity, and educational environments and black/white disparities in obesity: a complex systems simulation analysis. J Epidemiol Community Health.

[84] Georgeff M, Pell B, Pollack M, Tambe M, Wooldridge M, Muller JP, Rao AS, Singh MP (1999). Intelligent Agents V: Agents Theories, Architectures, and Languages.

[85] Norling EJ (2009). Modelling human behaviour with BDI agents.

[86] Kollingbaum MJ, Norman TJ, Dastani MM, Dix J, El Fallah-Seghrouchni A (2004). Programming Multi-Agent Systems.

[87] Cervero R, Denman S, Jin Y (2019). Network design, built and natural environments, and bicycle commuting: evidence from British cities and towns. Transport Policy.

[88] Stefansdottir H, Ninss P, Ihlebmk CM (2019). Built environment, nonmotorized travel and overall physical activity. Travel Behav Soc.

[89] Lu Y, Yang Y, Sun G, Gou Z (2019). Associations between overheadview and eye-level urban greenness and cycling behaviors. Cities.

[90] Marquet O, Floyd MF, James P (2020). Associations between worksite walkability, greenness, and physical activity around work. Environ Behav.

[91] Sarigiannis DA, Karakitsios SP, Antonakopoulou MP, Gotti A (2012). Science of the total environment exposure analysis of accidental release of mercury from compact fluorescent lamps (CFLs). Sci Total Environ.

[92] Musalaiah M, Venkata RP, Liyakhath AS, Hussain Z (2013). A review on theoretical air pollution dispersion models. Int J Pharm Chem Biol Sci.

[93] Sachdeva S, Baksi S, Siddiqui NA, Tauseef SM, Bansal K (2018). Advances in Health and Environment Safety Springer Transactions in Civil and Environmental Engineering.

[94] Stockie JM (2011). The mathematics of atmospheric dispersion modeling. SIAM Rev.

[95] Aumond P, Jacquesson L, Can A (2018). Probabilistic modeling framework for multisource sound mapping. Appl Acoust.

[96] Can A, Aumond P, Becarie C, Leclercq L (2019). Dynamic approach for the study of the spatial impact of road traffic noise at peak hours.

[97] Lesieur A, Aumond P, Mallet V, Can A (2019). Meta-modeling for urban noise mapping.

[98] Schreurs E, Jabben J, Verheijen E (2010). STAMINA-Model description: standard model instrumentation for noise assessment. Report 680740003/2010, tech.rep.RIVM.

[99] Anfosso-Ledee F, Paviotti M, Kephalopoulos S (2012). Common noise assessment methods in Europe (CNOSSOS-EU): to be used by the eu member states for strategic noise mapping following adoption as specified in the Environmental Noise Directive 2002/49/EC. Publications Office of the European Union.

[100] Xie C (2012). Interactive heat transfer simulations for everyone. Phys Teach.

[101] Oropeza-Perez I (2020). Simplified numerical model for analyzing the effects of the urban heat island upon low-rise buildings by using a free-license thermal simulation program. Urban Sci.

[102] Degraeuwe B, Thunis P, Clappier A (2017). Impact of passenger car NOX emissions on urban NO_2_ pollution—scenario analysis for 8 European cities. Atmos Environ.

[103] Efron B (1979). Bootstrap methods: another look at the jackknife. Ann Statist.

[104] Efron B, Tibshirani RJ (1993). An Introduction to the Bootstrap.

[105] Schulze J, Muller B, Groeneveld J, Grimm V (2017). Agent-based modelling of social-ecological systems: achievements, challenges, and a way forward. JASSS.

[106] Grimm V, Revilla E, Berger U (2005). Pattern-oriented modeling of agent-based complex systems: lessons from ecology. Science.

[107] Kaveh A, Kaveh A (2017). Advances in Metaheuristic Algorithms for Optimal Design of Structures.

[108] Liang F, Wong WH (2000). Evolutionary Monte Carlo: applications to CP model sampling and change point problem. Statist Sin.

[109] Bottolo L, Richardson S (2010). Evolutionary stochastic search for Bayesian model exploration. Bayesian Anal.

[110] Liquet B, Bottolo L, Campanella G, Richardson S, Chadeau-Hyam M (2016). R2GUESS: a graphics processing unit-based R package for Bayesian variable selection regression of multivariate responses. J StatSoft.

[111] Salle I, Yildizoglu M (2014). Efficient sampling and meta-modeling for computational economic models. Comput Econ.

[112] Lamperti F, Roventini A, Sani A (2017). Agent-based model calibration using machine learning surrogates. arXiv.

